# Interaction of Arsenic Species with Organic Ligands: Competitive Removal from Water by Coagulation-Flocculation-Sedimentation (C/F/S)

**DOI:** 10.3390/molecules24081619

**Published:** 2019-04-24

**Authors:** Muhammad Ali Inam, Rizwan Khan, Muhammad Akram, Sarfaraz Khan, Du Ri Park, Ick Tae Yeom

**Affiliations:** 1 Graduate School of Water Resources, Sungkyunkwan University (SKKU) 2066, Suwon 16419, Korea; aliinam@skku.edu (M.A.I.); rizwankhan@skku.edu (R.K.); enfl8709@skku.edu (D.R.P.); 2 Shandong Key Laboratory of Water Pollution Control and Resource Reuse, School of Environmental Science and Engineering, Shandong University, Qingdao 266200, China; m.akramsathio@mail.sdu.edu.cn; 3 Key Laboratory of the Three Gorges Reservoir Region Eco-Environment, State Ministry of Education, Chongqing University, Chongqing 400045, China; sfk.jadoon@yahoo.com

**Keywords:** arsenic, adsorption, coagulation-flocculation-sedimentation (C/F/S), competitive removal, organic ligands, interaction, water treatment

## Abstract

The co-occurrence of arsenic (As) and organic ligands in water bodies has raised environmental concerns due to their toxicity and adverse effects on human health. The present study aims to elucidate the influences of hydrophobic/hydrophilic organic ligands, such as humic acid (HA) and salicylic acid (SA), on the interactive behavior of As species in water. Moreover, the competitive removal behaviors of As(III, V) species and total organic carbon (TOC) were systematically investigated by coagulation-flocculation-sedimentation (C/F/S) under various aqueous matrices. The results showed the stronger binding affinity of As(V) than As(III) species, with a higher complexation ability of hydrophobic ligands than hydrophilic. The media containing hydrophilic ligands require smaller ferric chloride (FC) doses to achieve the higher As(III, V) removal, while the optimum FC dose required for As(III) removal was found to be higher than that for As(V). Moreover, hydrophobic ligands showed higher TOC removal than hydrophilic ligands. The pronounced adverse effect of a higher concentration of hydrophobic ligands on the removal efficiencies of As(V) and TOC was observed. The adsorption of As(V) on Fe precipitates was better fitted with the Langmuir model but the Freundlich isotherm was more suitable for As(III) in the presence of hydrophilic SA. Moreover, TOC removal was substantially decreased in the As(V) system as compared to the As(III) system due to the dissolution of Fe precipitates at higher As(V) concentrations. The results of FC composite flocs demonstrated that the combined effect of oxidation, charge neutralization and adsorption played an important role in the removal of both toxicants during the C/F/S process. In summary, the findings of the present study provide insights into the fate, mobility and competitive removal behavior of As(III, V) species and organic ligands in the water treatment process.

## 1. Introduction

In recent years, environmental contamination by arsenic (As) has attracted substantial attention, due to its ubiquity and acute toxicity to human health [1]. In the natural environment, it exists as As(III) and As(V) and leads to both long and persistent contamination when released into water [2]. Elevated concentrations of As in water bodies are mostly found in the vicinities of smelting, agriculture and mining sites [3]. For instance, the water streams in the Stampede and Slate Creek watersheds in the Kantishna Hills mining district in Alaska, USA, presented As contamination of around 720 µg/L [4]. High levels of As contamination (up to 7900 µg/L) have also been reported in the Tinto and Odiel Rivers in Spain and the Ashanti River in Ghana [5,6]. Moreover, As levels around 285 µg/L were found in the groundwater near abandoned antimony mines in Slovakia [7]. The potential adverse health effects of the oral uptake of such water-soluble As in the human body include gastrointestinal problems and carcinoma of the liver, lung, skin, bladder and kidney [8]. Therefore, World Health Organization (WHO) and United States Environmental Protection Agency (USEPA) have set the regulatory standard for As in drinking water at 10 µg/L [3].

Similar to arsenic, organic ligands are naturally ubiquitous on the surface and in groundwaters globally, produced as a result of different physical and geochemical processes [9,10]. The presence of organic ligands in water not only promotes microbiological growth in distribution networks but also increases the production of harmful disinfection by-products during water treatment [11,12]. In addition, the organic ligands may act as a carrier of various heavy metals as well as organic and inorganic pollutants and enhance their bioavailability by increasing their solubility in water [13,14,15]. Similarly, various hydrophobic/hydrophilic organic ligands such as humic acid (HA), fulvic acid (FA) and salicylic acid (SA) can also affect the transportation ability of As species in water. The organic ligands contain various reactive functional groups such as hydroxyl, phenolic and carboxylic acids, which can participate in the complexation and redox reactions with As, thus enhancing the fate, mobility and toxicity in an aqueous environment [16,17]. Therefore, removing both As and organic ligands from drinking water supplies is challenging and requires efficient water treatment technologies to reduce the associated toxicological risks to aquatic life and human health.

Among the many water treatment technologies available, the conventional coagulation-flocculation-sedimentation (C/F/S) is recognized as a cost-effective water treatment process. Several studies [10,18,19] have investigated the influence of organic ligands on the adsorption, solubility and mobility of As species in aqueous environments. Further, previous studies [10,20,21,22] have also reported the higher adsorption affinity of HA molecules for iron oxide than As species, which tends to change the surface properties of sorbent and compete with As species for active sorption sites, in turn increasing the mobility of As in water. The presence of HA also impedes the precipitation of ferric hydroxide at lower coagulant dosage due to the formation of soluble complexes with Fe^3+^ during the C/F/S process [23,24,25]. A recent study [17] exploring the complexation behavior of As with HA and FA in water found results indicating the increased binding affinity of organic ligands with As(V) as compared to with As(III). The previous relevant studies [9,26,27,28] were limited to the interactive behavior of As with hydrophobic organic ligands in water. Moreover, little is known about the interaction behavior between As species and hydrophobic/hydrophilic organic ligands during the drinking water treatment processes. To date, no study has explored the removal behaviors of As and hydrophobic/hydrophilic organic ligands, thus making it necessary to investigate the competitive removal of both toxicants during the C/F/S process.

Therefore, the aim of the present study is to explore the interactive behavior of As with hydrophobic/hydrophilic organic ligands with various As(III, V) concentrations. This work also evaluates the removals of As(III, V) and TOC by the conventional C/F/S process under varying (a) ferric chloride (FC) doses, (b) organic ligands concentrations and (c) As(III, V) concentrations. Moreover, the current work also examines the interaction and removal mechanism of As species in the complex aqueous environment.

## 2. Materials and Methods

### 2.1. Chemical Reagents and Stock Solutions Preparation

The reagent grade chemicals, that is, arsenic (III) oxide (As_2_O_3_), sodium arsenate dibasic heptahydrate (Na_2_HAsO_4_.7H_2_O), as well as the model organic ligands such as humic acid (HA) and salicylic acid (SA), were all purchased from Sigma-Aldrich (St. Louis, MO, USA); meanwhile, the analytical grade chemicals including FeCl_3_.6H_2_O (FC), HCl and NaOH were obtained from local suppliers. The stock solutions (100 mg L^−1^) of As(III) and As(V) were prepared by dissolving As_2_O_3_ and Na_2_HAsO_4_.7H_2_O in 1M NaOH and deionized water (DI) water, respectively. The 0.1 M FC stock solution was prepared by dissolving FeCl_3_.6H_2_O into DI water. The stock solutions of the organic ligands were prepared by adding 500 mg powder into 0.1 L DI water. The preparation methods used for HA and SA have been previously described in detail [29]. These model organic ligands that is, HA and SA had been widely used to simulate different water characteristics. Moreover, these model substances were obtained from commercial sources and have been widely used to provide consistent experimental conditions [29,30,31]. Prior to the binding and coagulation experiments, the total organic carbon (TOC) contents for HA and SA (10 mg L^−1^ each) were analyzed to be 4.17 and 2.40 mg C/L, respectively.

### 2.2. Experimental Procedures

In order to investigate the interactive behavior between (0.1–10 mgL^−1^) As(III, V) species and (10 mgL^−1^) organic ligands in water, the binding experiments were conducted at pH 7.0 ± 0.1. The glass vials were placed into a 360° rotator (TCLP-601P, Tainan, Taiwan) for 120 h in darkness with a rotating speed of 27 rpm at room temperature [17].

The C/F/S experiments were conducted using a jar tester equipment (Model SJ-10; Young Hana Tech Co., Ltd., Gyeongsangbuk-Do, Korea) on 100 mL samples in 250 mL beakers at 25 ± 1 °C. All experiments were performed at neutral pH 7.0 ± 0.1. The coagulation was initialized at 140 rpm rapid stirring for 3 min, after which flocculation was carried out with slow stirring at 40 rpm for 20 min [2,32]. The samples were then allowed to settle for 30 min, after which an aliquot of each sample was collected for TOC analysis using the TOC analyzer with an ASI-L liquid auto sampler (TOC-5000A, Shimadzu Corp, Kyoto, Japan). The remaining supernatant was filtered using a 0.45 µm glass fiber filter in order to analyze the residual As and Fe species using Inductively Coupled Plasma Optical Emission Spectrometry (ICP-OES: Model Varian, Agilent technologies, Sana Clara, CA, USA).

The experimental conditions for each set of C/F/S experiments are described as: (1) effect of FC dose (0.1 to 0.4 mM) on As(III, V) and TOC removal in the presence of 10 mgL^−1^ of HA/SA and 1 mg L^−1^ As(III, V); (2) influence of HA/SA concentration (1–20 mg L^−1^) on As(III, V) and TOC removal at various optimum FC doses and 1 mg L^−1^ As(III, V) and; (3) effect on As(III, V) and TOC removal in the presence of 10 mg L^−1^ of HA/SA and 0 to 10 mg L^−1^ As(III, V) concentrations and optimum FC doses. All of the experiments were conducted in duplicate and the average values of the results were reported.

### 2.3. Adsorption Isotherm Study

In order to further elucidate the adsorption capacity of As(III, V) species in the presence of HA/SA, experimental data and fitting parameters were acquired under different As(III, V) concentrations. The Langmuir and Freundlich models were used to elucidate the adsorption phenomena of both As species under different aqueous matrices. The nonlinear forms of the Langmuir and Freundlich adsorption models are presented as Equations (1) and (2), respectively:

(1) $$q_{e}\;=\;\frac{q_{m}K_{L}C_{e}}{1+{\;K}_{L}C_{e}}$$

(2) $$q_{e}\;=\;K_{F}C_{e}^{\frac{1}{n}}$$

where q_e_(mg.g^−1^) and q_m_(mg.g^−1^) are the equilibrium and maximum adsorption capacities, respectively, of As(III, V) on Fe surface sites; C_e_ (mgL^−1^) is the equilibrium concentration of As(III, V) in solution; K_L_(L.mg^−1^) in the Langmuir equation represent the Langmuir constant related to adsorption energy; and K_F_(mg.g^−1^) (L.mg^−1^)$\frac{1}{n}$ and *n* in the Freundlich equation represent the constants related to the adsorption capacity and intensity of heterogeneity, respectively.

### 2.4. Analytical Procedures

A pH meter (HACH: HQ40d Portable pH, Conductivity, oxidation-reduction potential (ORP) and ion selective electrode (ISE) Multi-Parameter Meter; Hach Company, Loveland, CO, USA) was used to adjust the pH of the solution. The free As species in solution was analyzed through high-performance liquid chromatography coupled with inductively coupled plasma mass spectrometry (LC-ICP-MS: Model Agilent 1100; Santa Clara, CA, USA). The organic ligand bound As was characterized by calculating the difference between total and free As species in water. The procedure was previously described in detail elsewhere [31,33]. Furthermore, the bonding features of pristine HA, SA and As(III, V) species as well as As-organic ligands composite complexes and FC composite flocs were determined using Fourier Transform Infrared Spectroscopy (FT/IR-4700, spectroscopy; JASCO Analytical Instruments, Easton, PA, USA).

## 3. Results and Discussion

### 3.1. As Binding to Organic Ligands

**Figure 1:**
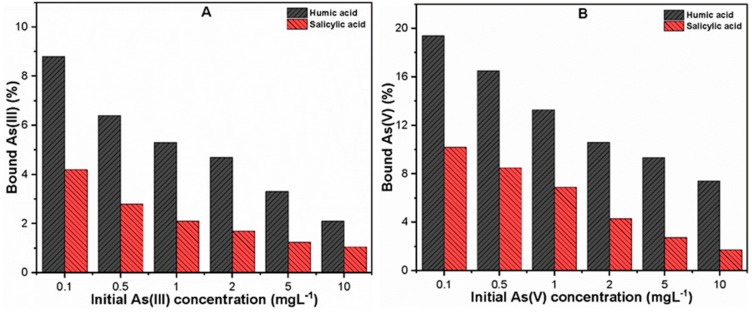
Percentages of bound (**A**) As(III) and (**B**) As(V) in 10 mgL^−1^ of humic/salicylic acid solutions under neutral pH (7 ± 0.1) at 25 ± 1 °C.

Figure 1 shows the percentage of organically bound As after the interaction of (0.1–10 mgL^−1^) As(III, V) species with 10 mgL^−1^ HA and SA. The results indicated a stronger binding affinity of As(V) than As(III) species along with better complexation ability with hydrophobic HA than hydrophilic SA. The percentages of As(III) and As(V) complexed with HA and SA were found to be (2.1–8.8% and 7.41–19.4%) and (1.05–4.02% and 1.71–10.2%), respectively. Our observations are consistent with the findings of a previous study [17] that As(III, V) may bind with organic ligands with different binding intensities. The presence of various functional groups in hydrophobic/hydrophilic organic ligands may be responsible for such discrepant binding behavior of As species with HA and SA [9,34]. The existence of a trace amount of cationic metals in organic substances may also contribute toward a great variability in binding affinity of As(III, V) with organic molecules via ternary binding mechanisms [28,35]. Moreover, the speciation of As may also influence the complexation ability, since inorganic As(III) and As(V) species at the pH studied here (7.0 ± 0.1) exist in the forms of As(OH)_3_ and (H_2_AsO_4_^−^, HAsO_4_^2−^), respectively [2]. The strong binding affinity of As(V) as compared to As(III) species suggests a potential stabilization effect of ligands molecules that occurs between the negatively charged As(V) and phenolate entity present in HA and SA [9]. This observation could also be attributable to the additional chelation by other functional groups present in organic ligands and H-bridges. Furthermore, Figure 1 also illustrates that the percent of bound As decreases with increasing initial As(III, V) concentration irrespective of the type of organic ligands. These results have the practical implication that, in natural water conditions, various types of organic ligands are present that may form dissolved complexes with As(III, V) due to the presence of various functional groups [17].

In general, the experimental results suggested that the binding affinity of As(III, V) species vary depending upon the characteristics of organic ligands present in water. Moreover, the presence of organic ligands may facilitate the transportation of As species to natural water sources, thereby enhancing the aquatic toxicity while also increasing human health risks. Therefore, further investigations were conducted in order to study the influences of hydrophobic and hydrophilic organic ligands on As and TOC removal using the conventional C/F/S process.

### 3.2. Effects of Organic Ligands on As(III, V) and TOC Removal by C/F/S

#### 3.2.1. Varying FC Dose

**Figure 2:**
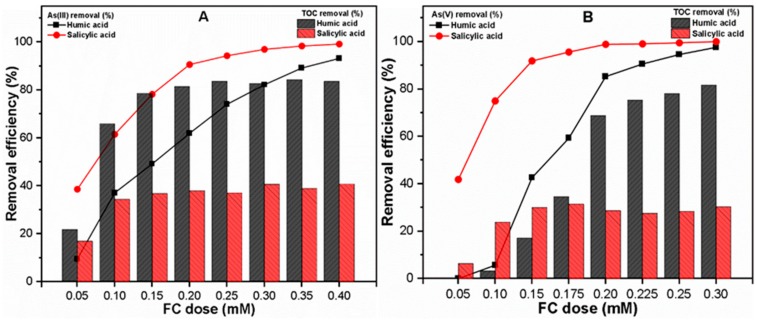
Under various ferric chloride (FC) doses, removal efficiencies of (**A**) As(III) and (**B**) As(V) and respective total organic carbon (TOC) from solution.

Figure 2 presents the influences of organic ligands on As and TOC removal at neutral pH under different FC doses (0.05–0.40 mM in the As(III) system) and (0.05–0.30 mM in the As(V) system). The results indicate that there is an insignificant effect of hydrophilic SA on As(III, V) removal under various FC doses. This may be related to the weaker acidic groups and the low molecular weight (LMW) compounds present in SA, thus leading to reduced interaction with Fe surface sites [36]. By contrast, significantly decreased removal efficiencies of both As(III, V) species under various FC doses were observed in the presence of hydrophobic HA molecules. These may be attributable to the fact that HA has a higher affinity for iron (Fe) surface sites than As, which may enhance the release of precipitated Fe in the solution, thus reducing precipitated Fe content for As removal in an aqueous environment [21,37]. In order to confirm whether Fe precipitation phenomena are involved in distinct removal behavior, Fe precipitation was also observed in both As(III, V) systems during the C/F/S process. The results showed a more adverse effect of hydrophobic HA on Fe precipitation in the As(V) system (Supplementary Figure S1B) than in the As(III) system (Supplementary Figure S1A). Our findings are consistent with those of previous studies [2,35] which suggested that a stronger inner sphere complexation of anionic ligands molecules with active Fe surface sites may enhance the Fe solubility in solution. In the current study, the As(V) system showed increased Fe solubility, which is related to its anionic ligand characteristics (H_2_AsO_4_^−^, HAsO_4_^2−^) and the presence of negatively charged organic ligand molecules at neutral pH condition [2,36,37,38,39].

Figure 2A shows 89.16% and 90.59% As(III) removal with the optimum doses (OD) of 0.35 and 0.20 mM FC in the presence of HA and SA, respectively. However, around 90.55% and 91.86% As(V) removal with OD of 0.225 and 0.15 mM FC was observed in the presence of HA and SA, respectively (Figure 2B). Note that a smaller FC dose is required in the As(V) system containing hydrophilic ligands. This may be attributable to the increased Fe precipitation in hydrophilic waters (Figure S1B) and strong adsorption affinity of As(V) species toward active Fe surface sites [2]. Similar to the As(V) removal behavior, the TOC removal was also strongly inhibited in hydrophobic waters containing As(V) species under different FC doses (Figure 2B). The TOC removals in the As(III) system at OD were found to be 91.59 and 41.18% in the presence of HA and SA ligands (Figure 2A), respectively, while the As(V) system presented 81.86 and 32.58% TOC removal, respectively (Figure 2B). The relatively lower TOC removal in the As(V) system than the As(III) system may be related to the fact that the FC dose required to achieve OD was less in the case of the As(V) system than the As(III) system. These results are consistent with those of earlier studies [40,41] which presented the higher removal of hydrophobic organic ligands as compared to hydrophilic ones during the removal process. Our findings suggested that the type of As species as well as the characteristics of organic ligands may influence the Fe precipitation phenomena and thereby affect the overall performance of the C/F/S process.

#### 3.2.2. Varying Organic Ligands Concentration

**Figure 3:**
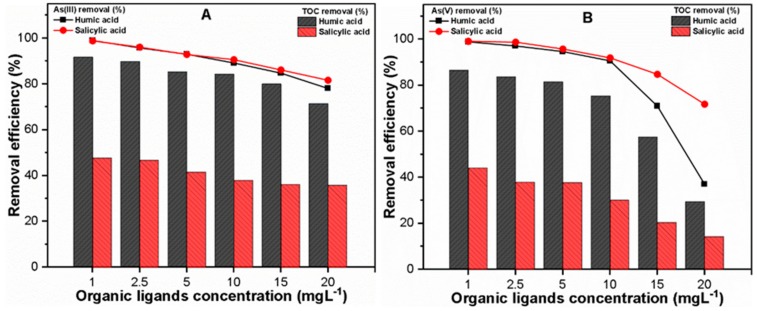
At various organic ligands concentrations, removal efficiencies of (**A**) As(III) and (**B**) As(V) and respective TOC from solution.

Figure 3 illustrates the effect of HA/SA (1–20 mgL^−1^) on the As(III, V) and TOC removal with the selected optimum FC dose of each system. As shown in Figure 3A, As(III) presented a similar removal trend irrespective of the type of organic ligand. For instance, the As(III) removals were observed to be 98.95 and 98.79% in the presence of 1 mgL^−1^ HA and SA, respectively. With an increase in the concentrations of both organic ligands (HA and SA) up to 20 mg L^−1^, the As(III) removal decreased to 78.04 and 81.62%, respectively (Figure 3A). The Fe precipitation was also observed during the coagulation process to explore the possible phenomena of reduced As(III) removal with increasing organic ligands concentration. The results indicated an insignificant effect on Fe precipitation in the As(III) system, even at higher concentrations (20 mg L^−1^) of both organic ligands (Supplementary Figure S2A), thus confirming the competitive inhibition effect of organic ligands molecules during As(III) removal. In addition, the organic ligands contain high binding affinity toward Fe precipitates and may thus enhance the As(III) solubility under coexisting environment [9,42]. Furthermore, the TOC removal was found to be in the ranges of 77.54–99.62% and 38.84–51.87% in the presence of (1–20 mg L^−1^) HA and SA, respectively (Figure 3A). The higher TOC removal in the presence of hydrophobic HA may be ascribed to the interaction of reactive anionic functional groups in HA with cationic Fe precipitates, thus forming insoluble complexes in water due to enhanced flocculation [43].

Compared with As(III), an increased reduction in As(V) removal was observed at HA/SA concentrations over 10 mgL^−1^, where a significant decline was found in the presence of HA (Figure 3B). The results indicated that 90.55 and 91.86% As(V) removal and 81.86 and 32.58% TOC removal were found in the presence of 10 mgL^−1^ HA and SA, respectively. Upon the addition of 20 mgL^−1^ of HA and SA in the As(V) system, around 30.97 and 71.76% As(V) and 31.88 and 15.42% TOC removal was achieved (Figure 3B). These remarkable declines in the removals of As(V) and TOC may be attributable to the significant reduction in Fe precipitation at higher organic ligands concentration (Supplementary Figure S2B). Moreover, strong electron donation by both negatively charged organic (HA or SA) and inorganic (H_2_AsO_4_^−^, HAsO_4_^2−^) ligands molecules to surficial Fe atom may have played a major role in the dissolution of Fe precipitates in this system [44]. The results indicated that extensive reductions in both As(V) and TOC removals were observed in the presence of a higher concentration of hydrophobic HA (Figure 3B). This might be related to the higher anionic binding affinity of HA with strong adsorption potential toward cationic Fe precipitates [43]. The addition of negatively-charged inorganic ligands such as HAsO_4_^−2^ may promote the formation of soluble complexes in an aqueous environment, thereby decreasing the amount of Fe precipitates required to remove both contaminants from water [2]. In general, these results suggested that water chemistry should be considered for the sustainable remediation of heavy metal pollution.

#### 3.2.3. Varying As(III, V) Concentration

**Figure 4:**
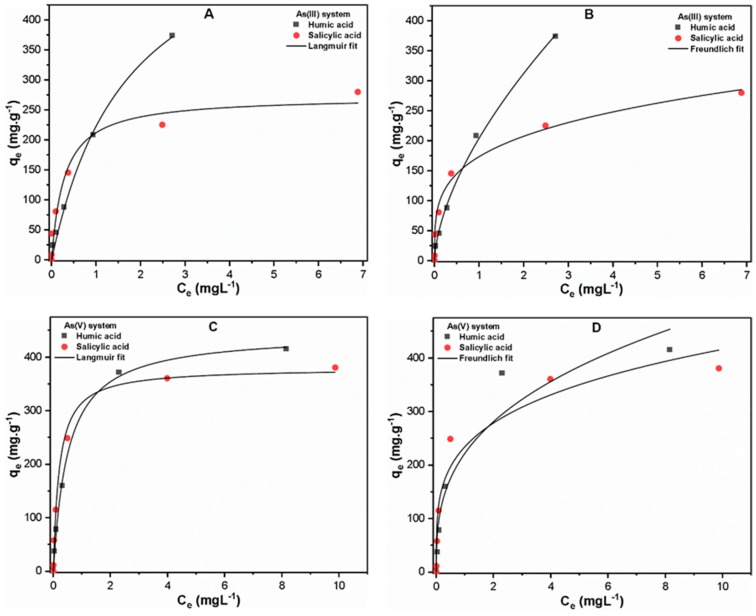
Adsorption isotherm of As adsorption onto precipitated Fe in (**A**,**B**) As(III); and (**C**,**D**) As(V) system in the presence of 10 mgL^−1^ organic ligands under varying initial As concentration (0–10 mgL^−1^) at pH 7.0 ± 0.1.

Figure 4 shows the experimental results of both the As(III, V) system and the Langmuir and Freundlich isotherm fittings under the influence of HA/SA at various initial As(III, V) concentrations (0–10 mgL^−1^). Interestingly, the isotherm study indicated a higher adsorption capacity of As(III) (608.36 mg.g^−1^) than As(V) (445.11 mg.g^−1^) in the presence of HA (Table 1). This may be attributable to the fact that the As(III) system presented an insignificant effect on Fe precipitation (Supplementary Figure S3A), while the Fe precipitation was significantly reduced at higher As(V) concentrations (Supplementary Figure S3B). Moreover, the reduced availability of Fe in the As(V) system impedes the attachment of As(V) and organic ligands complexes, thus resulting in less As(V) adsorption. Similar behavior was observed in the case of SA, where Fe precipitation was significantly decreased (Supplementary Figure S3B), resulting in less As(V) removal from solution. Moreover, the adsorption of As(V) onto Fe precipitates followed Langmuir isotherm, as depicted by their strong correlation coefficient (R^2^) (Table 1), hence confirming the monolayer formation with the same energy of adsorption for each Fe active site [32]. Besides significant decrease in Fe precipitation, the As(V) presented a higher adsorption capacity (379.87 mg.g^−1^) than As(III) (272.29 mg.g^−1^) in the presence of hydrophilic SA (Table 1). This may be due to the presence of LMW compounds in SA and strong adsorption potential of As(V) toward Fe precipitates [2,36]. The adsorption of As(III) in the presence of SA followed Freundlich isotherm, thus indicating multilayer adsorption onto Fe precipitates [45]. These results suggested that As(V) species are likely to affect the Fe precipitation phenomena and thereby decrease the overall treatment efficiency of the C/F/S process.

**Figure 5:**
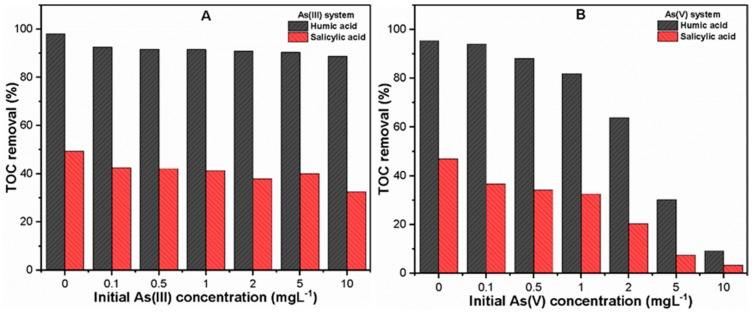
Under various initial As(III, V) concentrations (0–10 mgL^−1^), TOC removal in (**A**) As(III) and (**B**) As(V) system in the presence of 10 mg L^−1^ organic ligands (HA/SA).

Figure 5 shows the TOC removal for both As(III, V) systems under varying initial As(III, V) concentrations (0–10 mg L^−1^). The results indicate that there was higher TOC removal efficiency in the As(III) system than the As(V) system. The TOC removals observed at 1 mg L^−1^ As(V) were 81.86% and 32.58% in the presence of HA and SA, respectively. With increasing As(V) concentrations from 2 to 10 mg L^−1^, significant reductions in TOC removal (63.89 to 9.24)% and (20.39 to 3.30)% were observed in the solutions containing HA and SA, respectively. This may be attributable to the fact that Fe dissolution occurs at higher initial As(V) concentrations (Supplementary Figure S3B). However, the higher removal of hydrophobic HA as compared to that of SA is linked with their strong adsorption potential toward the Fe surface [46]. Our results are also consistent with previous studies [40,41] that reported the higher removal of hydrophobic organic ligands than hydrophilic ones during the C/F/S process. These results also suggested the competitive inhibition effect of hydrophobic organic ligands in the removal of heavy metals during C/F/S.

### 3.3. Interaction and Removal Mechanism

**Figure 6:**
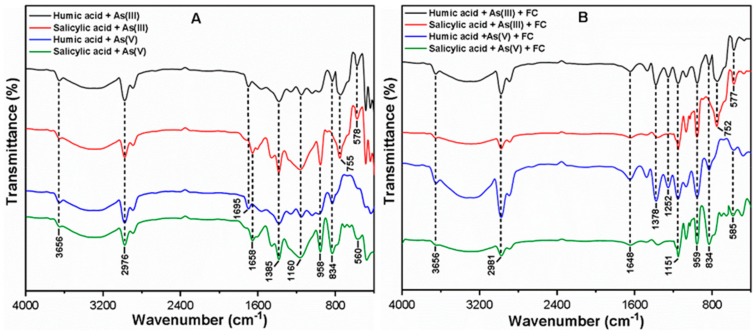
Fourier transform infrared (FT-IR) spectra of (**A**) As(III, V) species after interaction with organic ligands and (**B**) FC composite flocs after the C/F/S process.

The Fourier transform-infrared (FT-IR) spectra of As(III, V) species after the interaction with HA/SA are presented in Figure 6A. The peak appearing at ~1695 cm^−1^ in the presence of HA indicates that the As(III, V) species interacts with C=O groups to form metal ion complex [17]. The peaks observed in the range of 1400–900 cm^−1^ suggest the formation of complexes between As(III, V) and organic ligands through C=C stretch in the aromatic ring and OH bending in phenols [47]. The new band appearing at ~834 cm^−1^ indicates the complexation of As(V) species with organic ligands, while the peaks at ~755 and 578 cm^−1^ were ascribed to the binding of As(III) species (Supplementary Figure S4). Interestingly, the fraction of As(III) species was oxidized to As(V) as a result of the interaction with hydrophobic HA (Figure 6A), which may be attributable to the presence of the high molecular weight (HMW) organic compounds and oxygen-containing functional groups in HA [17,48]. Based on these observations, it may be concluded that the characteristics of organic ligands play a critical role in the transformation of As species in the natural waters.

Figure 6B shows the IR spectra of FC composite flocs after the reactions with As(III, V) species and organic ligands. The band appearing at ~1648 cm^−1^ was attributed to the attachment of As-organic ligands complexes onto Fe flocs through C=O stretching (amide) [50]. The shifts in the peaks to ~1378 and 1252 cm^−1^ correspond to the involvement of carboxylic, hydroxyl and phenolic groups in complex formation with As(III, V) via certain mechanisms such as ligand exchange and H-bridges [17,50]. Some of the new peaks that appeared at ~834 cm^−1^ and (~752 and 577 cm^−1^) in the IR spectra of FC composite flocs were ascribed to the adsorption of As(V) and As(III) species onto Fe precipitates, respectively (as confirmed from Supplementary Figure S4). In addition, the oxidation of As(III) to As(V) was observed in the presence of HA, as confirmed by the band appearing at ~834 cm^−1^ (Figure 6B). By contrast, the two broad bands appearing at ~752 and 577 cm^−1^ were observed in the presence of SA in the As(III) system, which further suggests the stretching of As(III)-O bonds onto Fe precipitates (Figure 6B). These findings are consistent with those of a previous study [17,48] that reported the oxidation of As(III) species in the presence of HA molecules via electron pair donation. Based on the experimental results and FT-IR analysis, the removal mechanism of As species could be a combination of oxidation, charge neutralization and adsorption. However, the removal mechanism in aquatic environment may change due to the characteristics of the organic ligands and As(III, V) species in the water environment.

## 4. Conclusions

In the current study, we examined the interactive behavior of As(III, V) species with hydrophobic/hydrophilic organic ligands (HA and SA) in an aqueous environment. Furthermore, we systematically investigated the competitive removal behavior of As(III, V) species and total organic carbon (TOC) by coagulation-flocculation-sedimentation (C/F/S) in a heterogeneous aqueous environment. Our study found that As(V) could form dissolved complexes more strongly with hydrophobic HA than hydrophilic SA. The solution containing hydrophobic ligands requires a higher FC dose to achieve higher As(III, V) removal, while the optimum FC dose required for As(V) removal was found to be less than that for As(III). In addition, lower TOC removal was observed in hydrophilic waters than hydrophobic waters, thus suggesting that there was less interaction of SA molecules with Fe surface sites. Distinct adverse effects on the removal of As(V) and TOC were observed in the presence of a higher concentration of hydrophobic ligands. The adsorption of As(V) onto the Fe surface indicated better fitting with the Langmuir model, thereby showing higher adsorption potential with both organic ligands. The mechanisms of oxidation, charge neutralization and adsorption may be involved in the removal of both pollutants. The findings of the current study may contribute to elucidating the fate, mobility and competitive removal behavior of As(III, V) species and organic ligands in a complex water environment.

## Figures and Tables

**Table 1:** Adsorption isotherm fitting parameters of As(III, V) species onto Fe precipitates in the presence of various organic ligands.

Organic Ligands	As Type	Langmuir Fitting			Freundlich Fitting		
		*K_L_* (L.mg^−1^)	*q_m_* (mg.g^−1^)	R^2^	*K_F_* (mg.g^−1^) (L.mg^−1^)$\frac{1}{n}$	*n*	R^2^
Humic acid	As(III)	0.579	608.36	0.995	204.72	1.64	0.997
	As(V)	1.954	445.11	0.998	222.54	2.94	0.933
Salicylic acid	As(III)	3.531	272.29	0.956	173.79	3.88	0.992
	As(V)	4.844	379.87	0.992	234.79	4.02	0.944

## Supplementary Materials

The following are available online at https://www.mdpi.com/1420-3049/24/8/1619/s1, **Figure S1.** (A) As(III) and; (B) As(V) system (1 mgL^−1^ As(III, V) concentration) showing Fe precipitation as a function of FC dose under neutral pH (7.0 ± 0.1) in the absence (Control) and presence of 10 mgL^−1^ humic/salicylic acid; **Figure S2.** At various organic ligands concentration (1–20 mg L^−1^), showing Fe precipitation in (A) As(III); and (B) As(V) system (1 mg L^−1^ As(III, V) concentration) under optimum FC doses at neutral pH (7.0 ± 0.1); **Figure S3.** At various As concentration (0–10 mg L^−1^) and humic/salicylic acid (10 mg L^−1^) showing Fe precipitation in (A) As(III) and; (B) As(V) system under optimum FC doses at neutral pH (7.0 ± 0.1); **Figure S4.** FT-IR spectra of arsenic (III) oxide, sodium arsenate dibasic heptahydrate, humic and salicylic acid powder. The references [17,50,52,53] are mentioned in the supplementary materials.
