# Supplementary material for: Interaction of Arsenic Species with Organic Ligands: Competitive Removal from Water by Coagulation-Flocculation-Sedimentation (C/F/S)
Source: Molecules. 2019 Apr 24;24(8):1619. doi: 10.3390/molecules24081619 (PMC6515111; doi:10.3390/molecules24081619)
Supplement: Supplementary file 1 [file molecules-24-01619-s001.pdf]

molecules

Supplementary Material

Interaction of Arsenic Species with Organic Ligands: Competitive Removal from Water by Coagulation-Flocculation-Sedimentation (C/F/S)

Muhammad Ali Inam[1], Rizwan Khan[1], Muhammad Akram[2], Sarfaraz Khan[3], Du Ri Park[1] and Ick Tae Yeom[1,*]

[1] Graduate School of Water Resources, Sungkyunkwan University (SKKU) 2066, Suwon 16419, Korea; aliinam@skku.edu (M.A.I.); rizwankhan@skku.edu (R.K.); enfl8709@skku.edu (D.R.P.)

[2] Shandong Key Laboratory of Water Pollution Control and Resource Reuse, School of Environmental Science and Engineering, Shandong University, Qingdao 266200, China; m.akramsathio@mail.sdu.edu.cn (M.A.)

[3] Key Laboratory of the Three Gorges Reservoir Region Eco-Environment, State Ministry of Education, Chongqing University, Chongqing 400045, China; Sfk.jadoon@yahoo.com (S.K.)

* Correspondence: yeom@skku.edu; Tel.: +82-31-299-6699

3. Results and Discussion

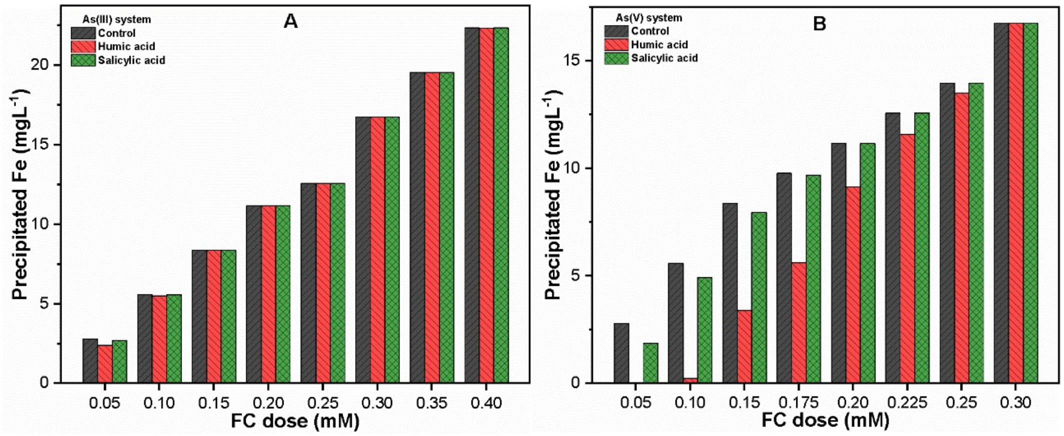

Figure S1. (A) As(III) and; **(B)** As(V) system (1 mgL^{-1} As(III,V) concentration) showing Fe precipitation as a function of FC dose under neutral pH (7.0 ± 0.1) in the absence (Control) and presence of 10 mgL^{-1} humic/salicylic acid.

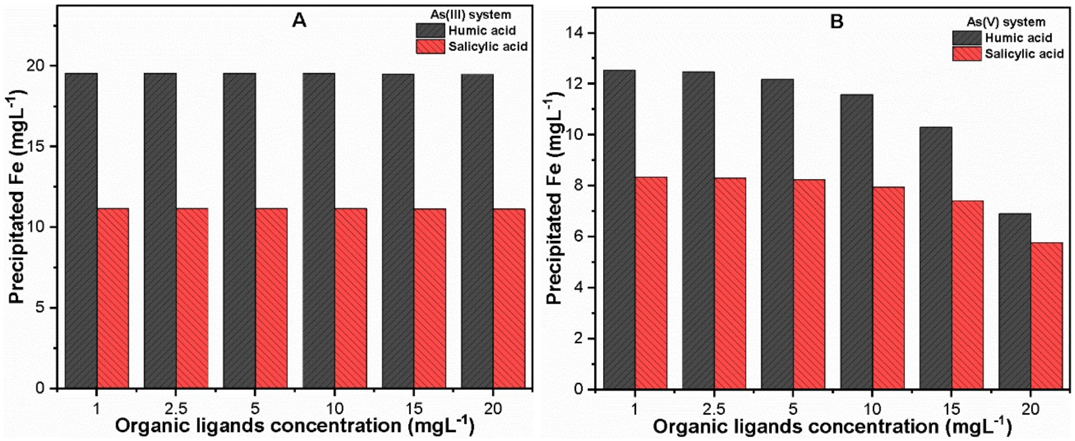

Figure S2. At various organic ligands concentration (1-20 mgL^{-1}), showing Fe precipitation in **(A)** As(III); and **(B)** As(V) system (1 mgL^{-1} As(III,V) concentration) under optimum FC doses at neutral pH (7.0 ± 0.1).

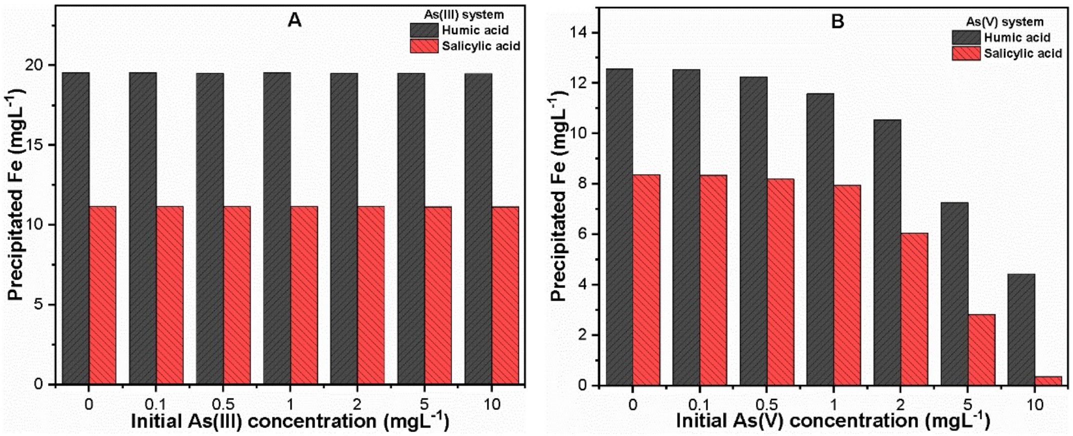

Figure S3. At various As concentration (0-10 mgL^{-1}) and humic/salicylic acid (10 mgL^{-1}) showing Fe precipitation in **(A)** As(III) and; **(B)** As(V) system under optimum FC doses at neutral pH (7.0 ± 0.1).

3.1. Fourier Transform Infrared Spectroscopy (FT-IR) of Powder Chemicals

The FT-IR analysis of powdered chemicals were recorded to expound the bond formation and functional groups as presented in Figure S4. The peaks around ~3655 and 2980 cm^{-1} corresponds to the partial N-H stretch and asymmetric stretching vibrations of C-H bond respectively [17,52]. The two small peaks at ~1654 and 1608 cm^{-1} was attributed to the symmetric and asymmetric stretching vibrations of C=O (COO^-) [50]. Moreover, the peaks in the range 1400–900 cm^{-1} corresponds to the enrichment of aliphatic or carbohydrate –OH functional groups in humic and salicylic acid [53]. The broad band observed at 832 cm^{-1} was attributed to As(V)-O stretching vibrations, while the two broad peaks that observed at 575 and 750 cm^{-1} was ascribed to the stretching vibration of As(III)-O bond [54]. Furthermore, the bands at ~674 and 555 cm^{-1} was subjected to the stretching vibrations of C-H and C-O-C groups respectively [17].

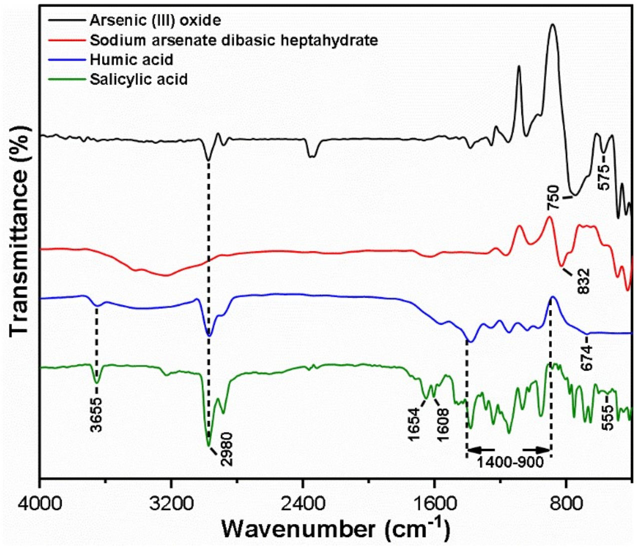

Figure S4. FT-IR spectra of arsenic (III) oxide, sodium arsenate dibasic heptahydrate, humic and salicylic acid powder.
